# Direct-Acting Oral Anticoagulant Therapy in Cancer Patients—A Review

**DOI:** 10.3390/cancers15102697

**Published:** 2023-05-10

**Authors:** Tomasz Górnicki, Kacper Bułdyś, Dorota Zielińska, Mariusz Chabowski

**Affiliations:** 1 Student Research Club No. 180, Faculty of Medicine, Wroclaw Medical University, 50-367 Wroclaw, Poland; tomasz.gornicki@student.umw.edu.pl (T.G.); 107253@student.uthrad.pl (K.B.); 2 Division of Histology and Embryology, Department of Human Morphology and Embryology, Wroclaw Medical University, 50-368 Wroclaw, Poland; 3 Department of Surgery, 4th Military Teaching Hospital, 50-981 Wroclaw, Poland; 4 Division of Anesthesiological and Surgical Nursing, Department of Nursing and Obstetrics, Faculty of Health Science, Wroclaw Medical University, 51-618 Wroclaw, Poland

**Keywords:** DOAC, LWMH, VKA, cancer, VTE

## Abstract

**Simple Summary:**

Direct-acting oral anticoagulants are becoming popular therapeutic option in patients with cancer. We investigated the available literature in order to try to sum up the information’s about them. In this article we present both, advantages and disadvantages of direct-acting oral anticoagulants. We found out that vitamin K antagonists are being slowly replaced by direct-acting oral anticoagulants, whereas compared to low-molecular-weight heparins they are mostly considered as an alternative option. Main concerns when using direct-acting oral anticoagulants are: a higher risk of major bleeding and drug-to-drug interactions with antineoplastic agents. We noticed that current guidelines of different scientific associations are not unanimous. We presented clinical trials on direct-acting oral anticoagulants in regard to cancer patients. Overall there are still a lot of discrepancies in field of by direct-acting oral anticoagulants therapy. In conclusion, this review supports the use of DOACs in various clinical situations.

**Abstract:**

Venous thromboembolism (VTE) is an important aspect in cancer patients. There are various pharmacological methods used for thrombotic event treatment. DOACs (direct-acting oral anticoagulants) are gaining popularity among both physicians and researchers and are slowly starting to replace VKAs (vitamin K antagonists), thus becoming a substitute or alternative option for LMWHs (low-molecular-weight heparins). In this article, we present DOACs’ main therapeutic advantages and disadvantages in patients with cancer. The only major concern with using DOACs is the higher risk of bleeding; however, there are discrepancies in this matter. There are still some types of cancer for which DOACs are not recommended. Specific cancer types may influence the efficacy of DOAC therapy. Additionally, race and ethnicity may affect therapy in cancer patients with DOACs. A sizeable number of clinical trials are focused on comparing DOACs with other anticoagulants. The current guidelines of different scientific associations are not unanimous in their DOAC assessments. There is still a need for more evidence of DOACs’ potential advantages over other methods of anticoagulation in cancer patients to facilitate their position in this recommendation. This literature review presents the current state of knowledge about the use of DOACs in patients with neoplastic growth.

## 1. Introduction

**Figure 1:**
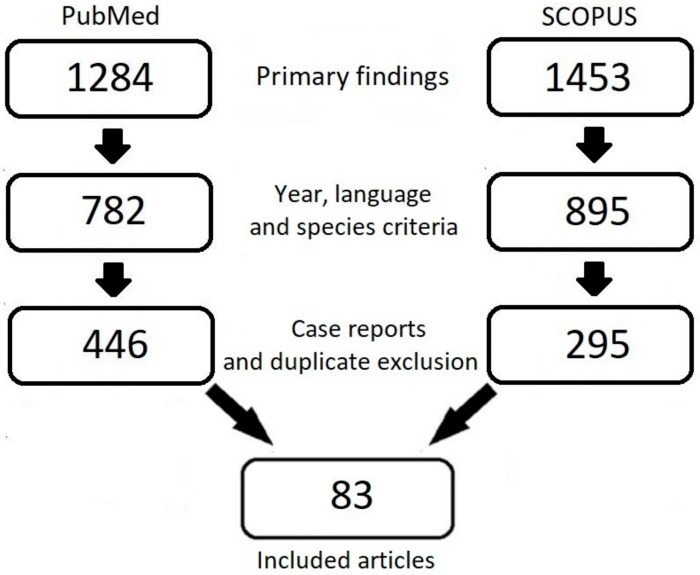
A schematic process for selecting publications for the review.

Venous thromboembolism (VTE) is a major concern for cancer patients. In patients with cancer, the overall risk of VTE is nine times higher than it is in the general population [1]. This correlation can be explained mostly by elevation of prothrombotic factor expression by either cancer cells or cells forming the tumor microenvironment [2,3]. These mechanisms are self-reinforcing processes because platelets present the ability to promote progression of the cancer [4,5]. Among molecular mechanisms of tumor cell-induced platelet aggregation (TCIPA), scientists described production of tissue factor (TF) by cancer cells [6], production and regulation of thrombopoietin (TPO) [7], and granulocyte colony stimulating factor [8]. Other mechanisms include adhesive protein production by cancer cells that allows for interaction with platelets; for example, P-selectin [9]. Variety of pro-thrombotic processes induced by the neoplastic process, combined with the perception of cancer as a chronic disease, elucidate the need for effective ways of VTE treatment and prophylaxis. Anticoagulant treatment is used to either prevent or treat thrombotic events. Therapeutic agents can be divided into two groups: parenteral and oral. The parenteral group includes unfractionated heparins (UFHs), low-molecular-weight heparins (LMWHs), fondaparinux, and bivalirudin, whereas oral agents are vitamin K antagonists (VKAs) and non-vitamin K antagonist direct oral anticoagulants (DOACs). UFHs have been mostly replaced with LMWHs, whereas VKAs are being replaced with DOACs. There is a growing body of evidence for DOAC use in cancer patients, e.g., in thromboprophylaxis and myeloproliferative neoplasms [10,11,12,13,14]. In a systematic review performed to assess the patients’ values and preferences regarding VTE, it was reported that disease-related health presents a broad spectrum of impact on patients’ lives. In the same report, it was highlighted that the need for therapy monitoring or dietary changes were not preferred [15]. Patients are concerned with the presence of cancer more than with VTE occurrence [16,17]. Receiving treatment with a lower possible interference with cancer treatment was highlighted [17]. Overall, oral medication is more desired over subcutaneous [15,16,18,19,20]. From patients’ perspective, the availability of a DOAC activity reversal agent was not a priority [20,21]. It is important to include the patient in the therapy decision-making process [15,16]. The aim of this review is to analyze the available literature on DOACs and their use in cancer patients. The article search was conducted in February 2023 within the PubMed and Scopus databases. To be included, the article (or at least the abstract) had to have been published in English, published after 2010, and carried out on humans. Case reports were excluded. The following combination of search terms was applied to find articles: ((cancer) OR (neoplasm)) and ((thrombosis) OR (VTE)) and ((NOAC*) OR (DOAC*) OR (LMWH*) OR (VKA) OR (VKAs)). A total of 446 articles in PubMed and 295 in SCOPUS were identified; after exclusion of duplicates and examining the abstract and/or full text, 83 articles remained (Figure 1). 

## 2. DOACs: Revolution in Anticoagulant Treatment

**Figure 2:**
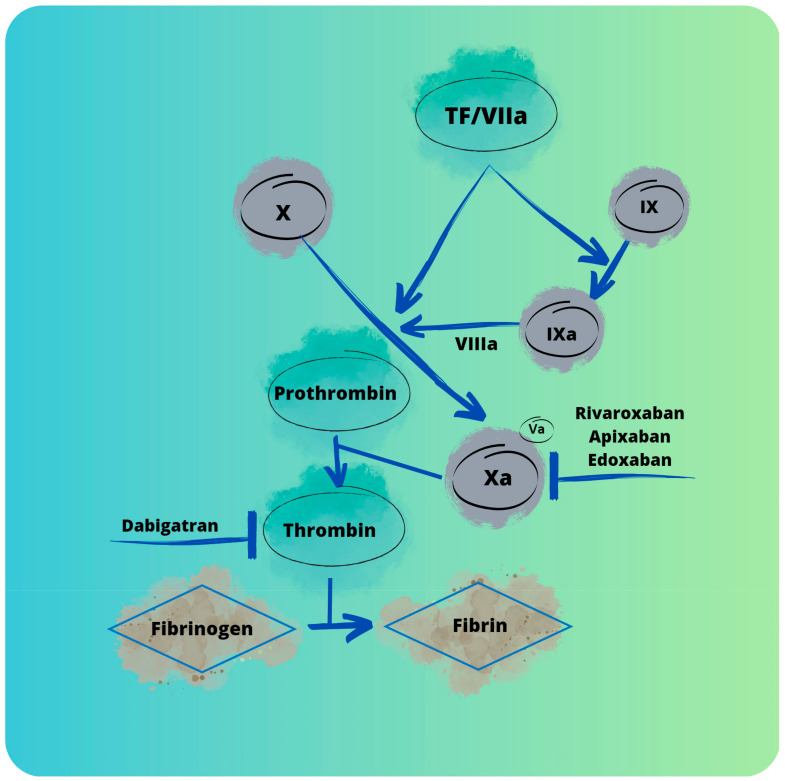
Visual representation of DOACs’ mechanisms of action presented on a model coagulation cascade.

NOACs (novel oral anticoagulants) are a group of anticoagulants that were introduced in October 2010 [22]. As time passed, the term “novel” became inappropriate, so to keep the acronym unchanged, its current meaning is non-vitamin K antagonist oral anticoagulants. This term was met with opposition from some members of the scientific community due to its lexical inaccuracy, leading to the creation of a new term: DOACs (direct-acting oral anticoagulants), proposed by the International Society on Thrombosis and Haemostasis in 2015. Other alternatives to the term present in the current scientific literature are target-specific oral anticoagulants (TSOACs), oral direct inhibitors (ODIs), and specific oral direct anticoagulants (SODAs) [23]. DOACs are the first line of anticoagulant treatment for many diseases. The importance of this group was confirmed by the WHO, which added DOACs to the World Health Organization’s Model List of Essential Medicines in 2019 [24]. Four main drugs belong to the group of DOACs: rivaroxaban, dabigatran, edoxaban, and apixaban. Their main mechanism of action is presented in Figure 2.

(A) Rivaroxaban is a direct inhibitor of factor Xa, binding directly to the active site of this factor [25]. It also has the ability to inhibit the activity of prothrombinase [26]. Rivaroxaban is a drug that is quickly absorbed after oral administration, with peak concentrations appearing after 2–4 h [27]. Current recommendations to use rivaroxaban include: prevention of atherothrombotic events in adult patients after an acute coronary syndrome with elevated cardiac biomarkers; prevention of atherothrombotic events in adult patients with coronary artery disease or symptomatic peripheral artery disease at a high risk of ischaemic events; prevention of VTE in adult patients undergoing elective hip or knee replacement surgery; treatment of deep vein thrombosis (DVT) and pulmonary embolism (PE); prevention of recurrent DVT and PE in adults; prevention of stroke and systemic embolism in adult patients with non-valvular atrial fibrillation (NVAF) with one or more risk factors; and treatment of VTE and prevention of VTE recurrence in children and adolescents under 18 years of age [28,29,30].
(B) Apixaban is a drug approved by the US Food and Drug Administration (FDA) in 2012. It is a highly selective inhibitor of factor Xa. It does not affect platelet aggregation. Its total bioavailability stands at about 50%, with peak plasma concentrations after 3–4 h [31]. Therapeutic indications of apixaban include: prevention of VTE events in adult patients who have undergone elective hip or knee replacement surgery; prevention of stroke and systemic embolism in adult patients with NVAF who have one or more risk factors; treatment of DVT and PE; and prevention of recurrent DVT and PE in adults [32,33,34].
(C) Edoxaban is a direct and specific inhibitor of factor Xa, with selectivity towards factor Xa nearly 10,000 times higher than thrombin [26]. Edoxaban was registered by the FDA in 2015. The highest concentration in plasma is noted after 1–2 h, with the half-life of the molecule being 10–12 h [35]. Currently, edoxaban is registered for the following therapeutic indications: prevention of stroke and systemic embolism in adult patients with NVAF with one or more risk factors; treatment of DVT and pulmonary embolism (PE); and prevention of recurrent DVT and PE in adults [36,37,38].
(D) Dabigatran etexilate is a factor IIa inhibitor. It is produced in the form of a prodrug that must be transformed into its active form by microsomal carboxylesterases in the liver. Due to its poor availability (around 6%), there is a need for the administration of high dabigatran dosages [39]. The half-life of dabigatran particles is 12–17 h, with the highest serum concentration achievable between 1 and 2 h after admission. It is important to mention that dabigatran is mainly eliminated by the kidneys; therefore, it is contraindicated in patients with renal failure [40]. Therapeutic indications of dabigatran include primary prevention of VTE events in adult patients who have undergone elective total hip or total knee replacement surgery; prevention of stroke and systemic embolism in adult patients with NVAF who have one or more risk factors; treatment of DVT and PE; and prevention of recurrent DVT and PE in adults [41,42,43].

## 3. DOACs’ Potential to Replace Classical VKAs in the Therapy of Patients with Cancer

VKAs were the first group of oral anticoagulant drugs used in therapy. They have been available since the 1950s. VKAs display their anticoagulant abilities by inhibiting vitamin K reductase, leading to the depletion of coagulation proteins (factors II, VII, IX, and X), which depend on vitamin K in their synthesis pathways [44]. Compared to DOACs, VKAs have various limitations, including slow onset and late offset of therapeutic activity. The dose of VKAs is not fixed, as in the case of DOACs, because it has to take into consideration the genetic polymorphism of patients, their metabolism, and their daily intake of vitamin K. This leads to the need for frequent monitoring of anticoagulation treatment, which is inconvenient for patients and costly for the health care system. In addition, the risk of bleeding is lower in DOACs compared to VKAs [12]. Studies have shown that DOACs constitute a group of drugs that overtake traditional VKAs in anticoagulation treatment [45,46,47]. Papers provide evidence of DOACs’ benefits compared to traditional therapy with VKAs [48,49,50,51,52,53,54]. In cancer patients, VKA use was associated with a higher risk of cancer-associated thrombosis (CAT) and recurrence of bleeding compared with the general population [55,56]. There is also a higher risk of VTE recurrence and anticoagulant-associated bleeding [53,57,58]. Administration of VKAs is also strained because of multiple drug–drug interactions in cancer patients. The major advantages of DOACs for patients with cancer in comparison to VKAs are predictable pharmacokinetics and pharmacodynamics, few drug–drug and drug–food interactions, and a wide therapeutic window [59]. Additionally, the persistence in DOACs’ usage is higher than that of VKAs [60]. Studies provide evidence that the administration of DOACs is associated with a lower risk of major bleeding (MB) in cancer patients with VTE. In contrast to DOACs, VKAs are also not recommended for acute VTE in patients with cancer [61,62,63,64,65,66]. Patients with active cancer treated with VKAs have a higher risk of death than patients treated with DOACs [67]. In patients with cancer and atrial fibrillation (AF), DOACs prove to be safer than traditional VKAs, leading to fewer incidences of MB, combined ischemic stroke/systemic embolism, intracranial bleeding, and major gastrointestinal bleeding [68]. In patients with AF and a history of cancer, DOACs seem to be safer than VKAs in patients aged < 75 years [69,70,71]. In partial contradiction to the data discussed in the previous statement, one of the studies analyzed states that in patients with gastrointestinal (GI) tract malignancies with and without comorbid AF, oral anticoagulant-related GI bleeding was more likely to appear in patients treated with DOACs in comparison to VKAs, especially in groups of older men [72,73]. The risk of bleeding is also reported to be higher in older men with genitourinary tract malignancies [73]. DOACs are postulated to decrease the risk of primarily DVT events in patients exposed to high-risk chemotherapy, with VKAs lacking this activity in a head-to-head comparison [74]. Patients with cancer using DOACs have a lower risk of ischemic stroke, systemic embolism, and myocardial infarction [75,76,77] compared to VKA users. A study concerning the anticoagulant treatment of post-stroke patients with cancer shows another example of DOACs’ higher value than traditional VKA treatment [78].

## 4. Could DOACs Be Used as a Substitute for Classic Anticoagulant LMWH Therapy?

Low-molecular-weight heparins (LMWHs) are considered a recommended treatment for VTE in cancer patients. They were introduced as an alternative to VKAs. LMHWs inhibit coagulation through the activation of antithrombin III, which binds to factor Xa and causes its inhibition. This results in the final common path not being activated. Xa inactivation means that prothrombin is not activated to thrombin, thereby not converting fibrinogen into fibrin for the formation of a clot [79,80]. The advantages of DOAC therapy are oral administration [62,75,81], rapid onset and offset of action [74,82,83,84,85], no need for therapy monitoring [81,82,83,86,87,88], patient comfort [61,83], lower therapy cost than LMWHs, fixed doses [83,84], few drug–drug or food–drug interactions, predictable anticoagulation effects, and the presence of specific reversal agents [81,82,88]. The disadvantages of DOACs are lower efficacy than LMWHs in vomiting patients, less clinical experience than LMWHs, and caution advised in renal insufficiency [82,89,90]. Additionally, there is a lack of studies in obese patients and those with platelet counts lower than 50,000 mg/L [90]. There is a concern in terms of monitoring DOACs’ anticoagulation effect, but UPLC–MS/MS is being proposed as a potential solution [91]. DOACs had a higher, yet nonsignificant, risk of MB compared with LMWHs [55]. However, it was reported that GI bleeding associated with the use of DOACs may be a cancer revelator [70]. It is important to assess patients’ risk of VTE occurrence, which can be done using the Khorana Risk Score (KRS). KRS is a point-based score used to assess the risk of VTE occurrence in ambulatory cancer patients. It divides the population into three risk categories: low, intermediate and high. It takes into account five parameters, which are: the type of cancer, with distinctions of high risk (pancreas and stomach) and low risk (lung, lymphoma, gynecologic, bladder, testicular); a prechemotherapy platelet count of 350 × 10^9^/L or more; a hemoglobin level of less than 100 g/L or the use of red-cell growth factors; a prechemotherapy leukocyte count of more than 11 × 10^9^/L; and a BMI of 35 kg/m^2^ or more [92,93]. A review performed by Lenihan DJ et al. provides evidence that DOACs are not inferior to LMWHs in terms of treatment of VTE. It was also noted that not every cancer patient should receive routine pharmacologic thromboprophylaxis, but those who are included in the high-risk group should be treated with either DOACs or LMWHs [92,93,94,95,96,97]. In a Cochrane meta-analysis, it was found that there were no differences between DOACs and conventional anticoagulation of recurrent VTE. However, the evidence was moderate or low [98]. When discontinuing the anticoagulant therapy, patients with a metastatic disease are at greater risk of VTE recurrence at 6 and 12 months; however, with regard to incidental VTE, the risk is lower in this time period [99]. RIETE registry’s data analysis displayed a more consistent risk of subsequent VTE events in cancer patients with superficial vein thrombosis (SVT) than in cancer patients with DVT during the first 3 months of anticoagulant therapy [100]. Studies on patients with hematological malignancies showed a lower bleeding or recurrence rate in the DOAC group compared to the LMWH group [101]. With regard to incidental PE, its single subsegmental type was not significantly associated with the risk of recurrent VTE, whereas the multiple type presented an increased risk of recurrent VTE [102]. A high risk of recurrence in patients with gynecological and pancreatic or hepatobiliary cancer was also reported. In the same report, there is a mention of ECOG status of 1 or greater being a risk for recurrence [103]. In another systematic review, DOACs were displayed as more effective than LMWHs in the prevention of recurrent VTE (rVTE), yet they were associated with an increased risk of MB and clinically relevant non-major bleeding (CRNMB) [104]. Rivaroxaban and LMWHs reduced the occurrence of peripherally inserted central venous catheter-related upper extremity VTE in cancer patients during chemotherapy [105]. In a randomized control trial (RCT) comparing rivaroxaban and enoxaparin in patients undergoing gynecological cancer surgery, it was proven that those agents had similar rates of thrombotic and bleeding events [106]. In a study conducted on the Asian population, similar rates of rVTE were reported when DOACs and enoxaparin treatment were compared. DOACs had similar VTE recurrence risk and MB at 12 months follow-up. The results of this study also suggest that there might be an association between race or ethnic differences and GI bleeding in treatments with DOACs in cancer-associated VTE (CAVTE) [107]. VTE recurrence was lower in patients treated with rivaroxaban than LMWHs [55,108]. In a systematic review on rivaroxaban and LMWHs, it was discovered that, compared to LMWHs, rivaroxaban has a lower risk of VTE recurrence, lower all-cause mortality, and a better overall NCB [56]. In a meta-analysis performed by Camilli M et al., the significant superiority of DOACs over LMWHs was reported in treating rVTE in cancer patients [109]. The major concern of DOACs therapy is their bleeding risk. In a phase III trial, edoxaban was reported to be noninferior to dalteparin in patients with CAVTE, with regard to the combined outcome of rVTE or MB [110]. DOACs treatment was associated with a lower risk of GI bleeding than enoxaparin. With regard to MB, there were no increased events. They were shown to have a lower rate of major GI bleeding when compared with enoxaparin [107]. DOACs are also preferred when anticoagulation therapy is going to be extended. In patients with CAT treated with DOACs, caution is required when patients are at higher risk of bleeding or drug-to-drug interactions [111]. Interestingly, in an observational study, the median time to a minor or MB occurrence was shorter in DOACs than LMWHs [85]. Xa inhibitors were associated with an increased overall risk of bleeding events when compared with LMWHs, with the majority of these events being CRNMB. The GI tract was reported to be the most common bleeding site. Additionally, the risk of intracranial bleeding while treating CAVTE was presented as a significant concern [112]. Contrary to Hussain MR et al.’s findings, the risk of MB was reported not to increase during DOAC treatment; however, CRNMB and GI bleeding risk were reported to be increased, which is consistent with Hussain MR et al.’s meta-analysis. What is more, there is a report that says there is no evidence of a significant difference between rivaroxaban and LMWHs in terms of MB. However, in the same study, taking CRNMB into account, there was a higher prevalence in the rivaroxaban group [56]. Data gathered in the meta-analysis of Mohamed MFH et al. showed the potential superiority of rivaroxaban concerning a lower mortality rate when compared with LMWHs; however, the result displayed marked heterogeneity [56]. Additionally, DOACs may be associated with a lower risk of intracranial hemorrhage than LMWHs with regard to metastatic brain disease and venous thrombosis [86]. When considering the safety of DOAC therapy, it was shown in a pilot study that extended treatment with DOACs seems to be associated with similar effectiveness and safety as LMWHs [85], as well as in a recent meta-analysis that supports the safety and efficacy of DOACs as a CAT treatment [113]. Furthermore, this therapy is comparable with LMWHs in active cancer patients undergoing treatment for cryptogenic ischemic stroke; however, the study was conducted on a small sample size [114]. In terms of GI tumors, DOACs show a comparable safety profile with LMWHs [109]. If anticoagulation treatment is required beyond 6 months, DOACs and LMWHs are safe to use; however, DOACs are associated with a higher risk of bleeding [115]. In another review, Xa inhibitors were shown to be comparable to LMWHs with regard to the risk of recurrent PEs. No significant difference was reported between Xa inhibitors and LMWHs in the risk of a fatal VTE; however, this was based on very low certainty evidence [112]. For patients who suffer from multiple myeloma and receive thalidomide- or lenalidomide-based regimens combined with chemotherapy and/or dexamethasone, as well as individuals with locally advanced or metastatic pancreatic cancer, the recommended anticoagulants are apixaban and rivaroxaban or LMWHs. Interestingly, VKAs are no longer recommended. In terms of secondary prevention of VTE, recent RCTs and meta-analyses indicated that apixaban, one of the DOACs, had the best safety and efficacy profiles in this category. DOACs are suggested over LMWHs or VKAs when dealing with patients undergoing chemotherapy for newly diagnosed NVAF, with the exception of patients with luminal gastrointestinal cancers with an intact primary or patients with active gastrointestinal mucosal abnormalities such as duodenal ulcers, gastritis, esophagitis, or colitis [116]. In another review, DOACs are shown to be a preferred agent in AF unless there are strong contraindications [117]. It is important that cytochrome P450 3A4-related drug-to-drug interaction of DOACs can interfere with their pharmacokinetics [61,90]. Some reports provide lists of chemotherapeutic agents and immunosuppressants that can potentially interfere with DOACs’ basin of known metabolic pathway activity [48,87]. Most of the time, drug–drug interactions occur between tyrosine kinase inhibitors [48,87,118]. Other groups are small-molecule inhibitors [119], monoclonal antibodies i.e., alemtuzumab, hormonal agents, i.e., enzalutamide, and immune-modulating agents, i.e., cyclosporine [118]. It is important to highlight that DOACs will interfere differently with antineoplastic agents, with apixaban having the lowest potential to interact with antineoplastic agents [120]. There is still a lot of research to be done to investigate possible drug-drug interactions. Specific cancer cells may inhibit the thrombin generation provided by apixaban or enoxaparin. The combined presence of BXCP3 or MCF7 cells and platelets was linked to a weaker impact on the antithrombotic effect of apixaban than enoxaparin. Interestingly, the presence of BXCP3 or MCF7 cells separately impacted the inhibitory strength of apixaban and enoxaparin on a similar level [121]. Although DOACs are promising therapeutic agents, it is not uncommon for LMWHs to be recommended over DOACs, e.g., for patients who present a high risk of bleeding, such as GI cancer patients, those who require frequent dose adjustments with chemotherapy-induced thrombocytopenia, those who receive ongoing anticancer therapies with potential drug–drug interactions, as well as those with brain metastases [113]. In Table 1, various treatment agents used in guidelines for treating thrombotic events in cancer patients are gathered.

Currently available guidelines are considering the role of DOACs in VTE prophylaxis among patients with cancers in three main settings. The first one being patients undergoing surgical treatment of malignancies with no distinction between classic and laparoscopic procedures. In this case, usage of DOACs is mostly not recommended [94,95,97], with the exception of apixaban, which is one of the recommended drugs in postoperative VTE prophylaxis [90,96]. In the case of hospitalized patients, DOACs are also not the recommended method of VTE prophylaxis [95,96,97]. Nevertheless, ASH Guidelines allow patients to continue using DOACs regimen when the prophylaxis was started before hospitalization [90]. The last group of patients widely discussed in the context of VTE prophylaxis are outpatients undergoing systemic chemotherapy or bone marrow transplantation. According to experts, VTE prophylaxis should be considered only in patients with high [90,94,95,96] or intermediate [97] risk of VTE (Khorana Risk Score two or higher). In this indication, DOACs are the recommended group of drugs alongside LMWH. It is important to mention that in patients with CVC, parenteral prophylaxis should be administered [94]. Recommendations discussed in the guidelines are reflected in conducted clinical trials. AVERT trail proved that thromboprophylaxis with apixaban reduced the risk of major VTE in most patients who were undergoing chemotherapy, same finding was made in subgroups of patients with gastrointestinal cancers, CVC presence, impaired renal function, and metastatic disease [124,125,126,127,128]. In the subgroup of patients initiating chemotherapy for recurrent disease, apixaban thromboprophylaxis was shown to be effective [129]. CASSINI study subgroups were analysed in field of thromboprophylaxis with rivaroxaban, and it was proved to be a viable therapeutic option [130,131]. In the pancreatic cancer subgroup, rivaroxaban was found to be potentially beneficial in VTE reduction [132]. However, in a placebo-controlled trial, no benefit of rivaroxaban treatment in high-risk ambulatory patients was displayed [133]. 

## 5. Clinical Trials

Presently, there are some clinical trials that are investigating DOACs in different setups. There is an ongoing study investigating whether drugs, inducing the CYP 3A4 isoenzyme of CYP450 and the P-gp transporter, significantly affect plasma levels of DOACs in patients with NVAF and VTE [134]. In this study, MACACOD DOACs are being investigated in terms of AF and rVTE [135]. In the CANVAS study, LMWHs and warfarin are compared to DOACs in cancer patients [136]. VICTORIE is a retrospective analysis of observational cohorts with VTE and active cancer or patients with VTE and a history of cancer who initiate anticoagulant treatment with a VKA, LMWHs, or DOACs [137]. Rivaroxaban’s anti-proliferative and other anti-cancer progression mechanisms are being assessed [138]. In OSCAR-SE, rivaroxaban is being compared with LMWHs [139]. A phase III RCT comparing rivaroxaban and placebo is being carried out [140]. Post-discharge prophylaxis with rivaroxaban in lung cancer patients is being investigated [141]. In VTE-POG, which is an ongoing open-label study of apixaban for VTE prevention in patients with newly diagnosed grade 4 glioma [142], there is a retrospective database analysis of health care claims data that investigates treating patients diagnosed with cancer and VTE with apixaban or LMWHs [143]. The ASTER study compares the effect of abelacimab relative to apixaban on VTE recurrence and bleeding in patients with CAVTE [144]. CASTA-DIVA investigated rivaroxaban compared with dalteparin in patients with CAT. Unfortunately, pre-defined criteria for noninferiority were not met because of an insufficient number of patients; however, its efficacy and safety results were consistent with those previously reported with DOACs [145]. The Caravaggio study reported noninferiority of oral apixaban to subcutaneous dalteparin treatment in CAT patients [146]. There is a report which sums up the role of DOACs in patients with CAT [147]. We did not identify ongoing studies that would include dabigatran or edoxaban as a single agent used in cancer patients.

## 6. Conclusions

Thrombotic events are a major concern in cancer patients, despite the constant efforts of physicians and scientists. The appearance of DOACs brought a new therapeutic option to the management of VTE. In this article, we showed that, in most cases, VKAs are being replaced with DOACs, whereas in terms of LMWHs, they are considered a substitute or alternative option. There are some major advantages to DOACs over VKAs, mainly fixed dosage and no need for therapy monitoring. The wide range of papers discussed in this review seem to support this statement in the therapy of patients with cancer. Nevertheless, there are still instances of VKAs superiority in comparison to DOACs that should not be omitted. Patients with GI and genitourinary tract malignancies, with particular emphasis on elderly men, are especially benefiting from VKAs therapy. A comparison between DOACs and LMWHs showed that their efficacy is predominantly comparable, although DOACs are more patient friendly in terms of their quality of life. It is also important to mention that there are still many uncertainties when it comes to DOACs usage in cancer patients with VTE. The main concerns revolve around the probable risk of MB and an insufficient amount of data and experience when it comes to treatment in obsessed patients and those with low plates count and renal insufficiency. Lack of strong evidence may lead to lower uptake of recent guidelines regarding prophylaxis with DOACs. Unfortunately, not every agent among the DOACs is equally researched. Rivaroxaban and apixaban are the main subjects of interest, with edoxaban and dabigatran being less popular in clinical trials. However, edoxaban was reported to be noninferior to subcutaneous dalteparin in the HOKUSAI-VTE study. The main concern when using DOACs is the higher risk of bleeding; however, there are some discrepancies between studies in this area. There is a possibility that ethnicity or race will be a field of particular interest for research, as it may affect the risk of bleeding. The presence of specific cancer cells may impact the efficacy of DOACs. Guidelines were found to vary in terms of therapeutic agents used in the management of thrombotic events. We identified some clinical trials that can contribute to standardizing the clinical approach for cancer patients. There is a need for further research on DOACs’ use in more specific situations. This review supports the use of DOACs in various clinical situations.

## Figures and Tables

**Table 1:** Current guidelines for thrombotic events and anticoagulation treatment in cancer patients.

Guidelines	SEOM 2019 [122]	THAZ 2019 [123]	ASCO 2020 [94]	ASH 2021 [97]	SGO 2021 [90]	NCCN 2021 [96]	ESMO 2023 [95]
Prophylaxis for VTE in hospitalized patients with cancer	-	-	Routine pharmacologic thromboprophylaxis may be offered	LMWHs	LMWHs	LMWHs, fondaparinux, UFHs	LMWHs, apixaban, rivaroxaban
Prophylaxis for VTE in ambulatory patients with cancer during systemic therapy	-	-	Routine pharmacologic thromboprophylaxis should not be offered. In high-risk patients, apixaban, rivaroxaban or LMWHs	LMWHs, fondaparinux	Rivaroxaban, apixaban, LMWHs	Apixaban, rivaroxaban, dalteparin and enoxaparin	-
Prophylaxis in patients with cancer undergoing surgery	-	-	Prophylaxis should be initiated preoperatively. LMWHs, UFHs	Prophylaxis should be initiated postoperatively. LMWHs	LMWHs, UFHs, apixaban	Apixaban, dalteparin and enoxaparin	LMWHs, UFHs
Prevention of rVTE	-	-	LMWHs, UFHs, fondaparinux or rivaroxaban.	-	-	-	-
Initial CAT treatment	LMWHs, rivaroxaban, UFHs, fondaparinux	-	-	LMWHs	-	-	LMWHs, UFHs, fondaparinux, apixaban, rivaroxaban
Short-term treatment for patients with active cancer	-	-	-	DOACs, LMWHs	-	-	-
Long-term treatment for patients with active cancer	LMWHs, DOACs	-	-	DOACs, LMWHs	LMWHs, apixaban, edoxaban or rivaroxaban	-	LMWHs, apixaban, edoxaban, rivaroxaban
CVCAT	LMWHs, DOACs	-	-	-	-	-	-
Incidental VTE	LMWHs, DOACs	Rivaroxaban, apixaban, dabigatran, warfarin, LMWHs	-	-	-	-	LMWHs, UFHs, fondaparinux
Recurrent VTE during anticoagulation therapy	LMWHs, DOACs	-	-	LMWHs	-	-	-
Central nervous system primary tumors and metastasis	LMWHs, DOACs	-	-	-	-	-	-
Anticoagulation in the absence of VTE to improve survival in cancer patients	Anticoagulant use in cancer patients should not be prescribed to improve survival	-	Anticoagulant use is not recommended to improve survival in patients with cancer without VTE	-	-	-	-

## Data Availability

The data that support the findings of this study are available from the corresponding author upon reasonable request.

## References

[1] Mulder F.I., Horvàth-Puhó E., van Es N., Van Laarhoven H.W.M., Pedersen L., Moik F., Ay C., Büller H.R., Sørensen H.T. (2021). Venous thromboembolism in cancer patients: A population-based cohort study. Blood.

[2] Pastori D., Cormaci V.M., Marucci S., Franchino G., Del Sole F., Capozza A., Fallarino A., Corso C., Valeriani E., Menichelli D. (2023). A Comprehensive Review of Risk Factors for Venous Thromboembolism: From Epidemiology to Pathophysiology. Int. J. Mol. Sci..

[3] Santoro C., Capone V., Canonico M.E., Gargiulo G., Esposito R., Sanna G.D., Parodi G., Esposito G. (2021). Single, dual, and triple antithrombotic therapy in cancer patients with coronary artery disease: Searching for evidence and personalized approaches. Seminars in Thrombosis and Hemostasis.

[4] Li X., Chen X., Gong S., Zhao J., Yao C., Zhu H., Xiao R., Qin Y., Li R., Sun N. (2023). Platelets promote CRC by activating the C5a/C5aR1 axis via PSGL-1/JNK/STAT1 signaling in tumor-associated macrophages. Theranostics.

[5] Mezouar S., Frère C., Darbousset R., Mege D., Crescence L., Dignat-George F., Panicot-Dubois L., Dubois C. (2016). Role of platelets in cancer and cancer-associated thrombosis: Experimental and clinical evidences. Thromb. Res..

[6] Gomes F.G., Sandim V., Almeida V.H., Rondon A.M., Succar B.B., Hottz E.D., Leal A.C., Verçoza B.R.F., Rodrigues J.C.F., Bozza P.T. (2017). Breast-cancer extracellular vesicles induce platelet activation and aggregation by tissue factor-independent and -dependent mechanisms. Thromb. Res..

[7] Stone R.L., Nick A.M., McNeish I.A., Balkwill F., Han H.D., Bottsford-Miller J., Rupaimoole R., Armaiz-Pena G.N., Pecot C.V., Coward J. (2012). Paraneoplastic thrombocytosis in ovarian cancer. N. Engl. J. Med..

[8] Suzuki A., Takahashi T., Nakamura K., Tsuyuoka R., Okuno Y., Enomoto T., Fukumoto M., Imura H. (1992). Thrombocytosis in patients with tumors producing colony-stimulating factor. Blood.

[9] Mege D., Aubert M., Lacroix R., Dignat-George F., Panicot-Dubois L., Dubois C. (2019). Involvement of Platelets in Cancers. Seminars in Thrombosis and Hemostasis.

[10] Laslo C.L., Bacalbasa N., Stanescu A.M.A., Carsote M., Bungau S., Rus M., Bratu O.G., Diaconu C.C. (2020). New oral anticoagulants—Possible extension to other indications (Review). Exp. Ther. Med..

[11] Hong K.S. (2019). Non-Vitamin K Antagonist Oral Anticoagulants in Medical Conditions at High Risk of Thromboembolism beyond Atrial Fibrillation. J. Stroke.

[12] Weitz J.I., Harenberg J. (2017). New developments in anticoagulants: Past, present and future. Thromb. Haemost..

[13] Lee L.H. (2016). DOACs—Advances and limitations in real world. Thromb. J..

[14] Weronska A., Papuga-Szela E., Broniatowska E., Undas A. (2021). Nonvitamin K Antagonist Oral Anticoagulant in Patients With Venous Thromboembolism and Polycythemia Vera or Essential Thrombocythemia: A Cohort Study. J. Cardiovasc. Pharmacol..

[15] Etxeandia-Ikobaltzeta I., Zhang Y., Brundisini F., Florez I.D., Wiercioch W., Nieuwlaat R., Begum H., Cuello C.A., Roldan Y., Chen R. (2020). Patient values and preferences regarding VTE disease: A systematic review to inform American Society of Hematology guidelines. Blood Adv..

[16] Noble S., Matzdorff A., Maraveyas A., Holm M.V., Pisa G. (2015). Assessing patients’ anticoagulation preferences for the treatment of cancer-associated thrombosis using conjoint methodology. Haematologica.

[17] Overvad T.F., Larsen T.B., Søgaard M., Albertsen I.E., Ording A.G., Noble S., Højen A.A., Nielsen P.B. (2020). Cancer-associated venous thromboembolism and the non-vitamin K antagonist oral anticoagulants: A review of clinical outcomes and patient perspectives. Expert Rev. Cardiovasc. Ther..

[18] Hutchinson A., Rees S., Young A., Maraveyas A., Date K., Johnson M.J. (2019). Oral anticoagulation is preferable to injected, but only if it is safe and effective: An interview study of patient and carer experience of oral and injected anticoagulant therapy for cancer-associated thrombosis in the select-d trial. Palliat. Med..

[19] Picker N., Lee A.Y., Cohen A.T., Maraveyas A., Beyer-Westendorf J., Mantovani L.G., Abdelgawwad K., Fatoba S., Thate-Waschke I.M., Bach M. (2021). Anticoagulation Treatment in Cancer-Associated Venous Thromboembolism: Assessment of Patient Preferences Using a Discrete Choice Experiment (COSIMO Study). Thromb. Haemost..

[20] Lanéelle D., Le Brun C., Mauger C., Guillaumat J., Le Pabic E., Omarjee L., Mahé G., SFMV VTE Study Group (2021). Patient Characteristics and Preferences Regarding Anticoagulant Treatment in Venous Thromboembolic Disease. Front. Cardiovasc. Med..

[21] Moyer G.C., Bannow B.S., Thornburg C., Rosovsky R., Wang T.F., Woller S., Thornhill D., Kreuziger L.B. (2018). Venous Thromboembolism: A Survey of Oral Anticoagulant Preferences in the Treatment of Challenging Patient Populations. Clin. Appl. Thromb. Hemost..

[22] Zhu J., Alexander G.C., Nazarian S., Segal J.B., Wu A.W. (2018). Trends and Variation in Oral Anticoagulant Choice in Patients with Atrial Fibrillation, 2010–2017. Pharmacotherapy.

[23] Shah S.B., Pahade A., Chawla R. (2019). Novel reversal agents and laboratory evaluation for direct-acting oral anticoagulants (DOAC): An update. Indian J. Anaesth..

[24] Di Cesare M., Jarvis J.D., Scarlatescu O., Leng X., Zaidel E.J., Burrone E., Eiselé J.L., Prabhakaran D., Silwa K. (2020). NOACs Added to WHO’s Essential Medicines List: Recommendations for Future Policy Actions. Global Heart.

[25] Roehrig S., Straub A., Pohlmann J., Lampe T., Pernerstorfer J., Schlemmer K.H., Reinemer P., Perzborn E. (2005). Discovery of the novel antithrombotic agent 5-chloro-N-([(5S)-2-oxo-3-[4-(3-oxomorpholin-4-yl)phenyl]-1, 3-oxazolidin-5-yl]methyl)thiophene-2-carboxamide (BAY 59–7939): An oral, direct Factor Xa inhibitor. J. Med. Chem..

[26] Samama M.M. (2011). The mechanism of action of rivaroxaban-an oral, direct Factor Xa inhibitor-Compared with other anticoagulants. Thromb. Res..

[27] Kubitza D., Becka M., Voith B., Zuehlsdorf M., Wensing G. (2005). Safety, pharmacodynamics, and pharmacokinetics of single doses of BAY 59-7939, an oral, direct factor Xa inhibitor. Clin. Pharmacol. Ther..

[28] European Medicines Agency: Xarelto (2023). European Medicines Agency. https://www.ema.europa.eu/en/medicines/human/EPAR/xarelto#authorisation-details-section.

[29] Janssen Pharmaceuticals, Inc Xarelto (Rivaroxaban) Prescribing Information. Revised 02/2023. https://www.janssenlabels.com/package-insert/product-monograph/prescribing-information/XARELTO-pi.pdf.

[30] Xarelto: EPAR-Product Information. Last Updated 23/02/2023. https://www.ema.europa.eu/en/documents/overview/xarelto-epar-medicine-overview_en.pdf.

[31] Agrawal A., Kerndt C.C., Manna B. (2022). Apixaban.

[32] European Medicines Agency: Eliquis (2023). European Medicines Agency. https://www.ema.europa.eu/en/medicines/human/EPAR/eliquis.

[33] Eliquis Prescribing Information. Revised 04/2021. https://www.accessdata.fda.gov/drugsatfda_docs/label/2021/202155s032lbl.pdf.

[34] Eliquis: EPAR-Product Information. Last Updated 09/09/2022. https://www.ema.europa.eu/en/documents/product-information/eliquis-epar-product-information_en.pdf.

[35] Padda I.S., Chowdhury Y.S. (2022). Chowdhury. Edoxaban.

[36] European Medicines Agency: Lixiana (2023). European Medicines Agency. https://www.ema.europa.eu/en/medicines/human/EPAR/lixiana.

[37] Savaysa Prescribing Information. Revised 01/2015. https://www.accessdata.fda.gov/drugsatfda_docs/label/2015/206316lbl.pdf.

[38] Lixiana: EPAR-Product Information. Last Updated 23/04/2021. https://www.ema.europa.eu/en/documents/product-information/lixiana-epar-product-information_en.pdf.

[39] Schwarb H., Tsakiris D.A. (2016). New Direct Oral Anticoagulants (DOAC) and Their Use Today. Dent. J..

[40] Nagarakanti R., Ellis C.R. (2012). Dabigatran in clinical practice. Clin. Ther..

[41] European Medicines Agency: Pradaxa (2023). European Medicines Agency. https://www.ema.europa.eu/en/medicines/human/EPAR/pradaxa.

[42] Pradaxa Prescribing Information. Revised 06/2021. https://www.accessdata.fda.gov/drugsatfda_docs/label/2021/214358s000lbl.pdf.

[43] Pradaxa: EPAR-Product Information. Last Updated 25/07/2022. https://www.ema.europa.eu/en/documents/product-information/pradaxa-epar-product-information_en.pdf.

[44] Baglin T., Bennett P.N., Brown M.J., Sharma P. (2012). Chapter 29—Drugs and haemostasis. Clinical Pharmacology.

[45] Van den Heuvel J.M., Hövels A.M., Büller H.R., Mantel-Teeuwisse A.K., De Boer A., Maitland-Van Der Zee A.H. (2018). NOACs replace VKA as preferred oral anticoagulant among new patients: A drug utilization study in 560 pharmacies in The Netherlands. Thromb. J..

[46] Lee S.I., Sayers M., Lip G.Y.H., Lane D.A. (2015). Use of non-vitamin K antagonist oral anticoagulants in atrial fibrillation patients: Insights from a specialist atrial fibrillation clinic. Int. J. Clin..

[47] Mocek A., Weber V., Schmölders J., Witt H., Gothe H. (2022). Preferences for and use of oral anticoagulants for stroke prevention in atrial fibrillation under real-world conditions in Germany: A survey among physicians. Prev. Med. Rep..

[48] Ravikumar R., Lim C.S., Davies A.H. (2017). The Role of New Oral Anticoagulants (NOACs) in Cancer Patients. Adv. Exp. Med. Biol..

[49] Vedovati M.C., Germini F., Agnelli G., Becattini C. (2015). Direct oral anticoagulants in patients with VTE and cancer: A systematic review and meta-analysis. Chest.

[50] Larsen T.B., Nielsen P.B., Skjøth F., Rasmussen L.H., Lip G.Y. (2014). Non-vitamin K antagonist oral anticoagulants and the treatment of venous thromboembolism in cancer patients: A semi systematic review and meta-analysis of safety and efficacy outcomes. PLoS ONE.

[51] van der Hulle T., den Exter P.L., Kooiman J., van der Hoeven J.J., Huisman M.V., Klok F.A. (2014). Meta-analysis of the efficacy and safety of new oral anticoagulants in patients with cancer-associated acute venous thromboembolism. J. Thromb. Haemost..

[52] Ebner M., Lankeit M. (2020). Antithrombotische Therapie bei Lungenembolie [Antithrombotic Treatment of Pulmonary Embolism]. Dtsch. Med. Wochenschr..

[53] Yan Y.D., Zhang C., Shen L., Su Y.J., Liu X.Y., Wang L.W., Gu Z.C. (2018). Net Clinical Benefit of Non-vitamin K Antagonist Oral Anticoagulants for Venous Thromboembolism Prophylaxis in Patients with Cancer: A Systematic Review and Trade-Off Analysis from 9 Randomized Controlled Trials. Front Pharmacol..

[54] Malavasi V.L., Vitolo M., Proietti M., Diemberger I., Fauchier L., Marin F., Nabauer M., Potpara T.S., Dan G.A., Kalarus Z. (2022). Impact of malignancy on outcomes in European patients with atrial fibrillation: A report from the ESC-EHRA EURObservational research programme in atrial fibrillation general long-term registry. Eur. J. Clin. Investig..

[55] Wumaier K., Li W., Cui J. (2022). New Oral Anticoagulants Open New Horizons for Cancer Patients with Venous Thromboembolism. Drug Des. Devel. Ther..

[56] Mohamed M.F.H., ElShafei M.N., Ahmed M.B., Abdalla L.O., Ahmed I., Elzouki A.N., Danjuma M.I. (2021). The Net Clinical Benefit of Rivaroxaban Compared to Low-Molecular-Weight Heparin in the Treatment of Cancer-Associated Thrombosis: Systematic Review and Meta-Analysis. Clin. Appl. Thromb. Hemost..

[57] Prandoni P., Lensing A.W., Piccioli A., Bernardi E., Simioni P., Girolami B., Marchiori A., Sabbion P., Prins M.H., Noventa F. (2002). Recurrent venous thromboembolism and bleeding complications during anticoagulant treatment in patients with cancer and venous thrombosis. Blood.

[58] Zhang J., Xu J., Zhang W., Jiang M., Liu J., Xu L., Liu G., Zhao Z. (2019). Quality Appraisal of Guidelines on Cancer-Associated Thrombosis Using AGREE II Instrument and Analysis of Current Status of New Oral Anticoagulants. Clin. Appl. Thromb. Hemost..

[59] Mekaj Y.H., Mekaj A.Y., Duci S.B., Miftari E.I. (2015). New oral anticoagulants: Their advantages and disadvantages compared with vitamin K antagonists in the prevention and treatment of patients with thromboembolic events. Ther. Clin. Risk Manag..

[60] Kim H., Lee Y.S., Kim T.H., Cha M.J., Lee J.M., Park J., Park J.K., Kang K.W., Shim J., Uhm J.S. (2020). A prospective survey of the persistence of warfarin or NOAC in nonvalvular atrial fibrillation: A comparison study of Drugs for symptom control and complication prevention of Atrial Fibrillation (CODE-AF). Korean J. Intern. Med..

[61] Iorga R.A., Bratu O.G., Marcu R.D., Constantin T., Mischianu D.L.D., Socea B., Gaman M.A., Diaconu C.C. (2019). Venous thromboembolism in cancer patients: Still looking for answers. Exp. Ther. Med..

[62] Dong S., Zhang Y., Li Y., Li Y., Miao Y., Zhao R., Zhai S. (2021). Direct Oral Anticoagulant for the Treatment of VTE in Cancer Patients: A Systematic Review and Meta-analysis. Ann. Pharmacother..

[63] Raskob G.E., van Es N., Segers A., Angchaisuksiri P., Oh D., Boda Z., Lyons R.M., Meijer K., Gudz I., Weitz J.I. (2016). Edoxaban for venous thromboembolism in patients with cancer: Results from a non-inferiority subgroup analysis of the Hokusai-VTE randomised, double-blind, double-dummy trial. Lancet Haematol..

[64] Karakatsanis S.J., Roumpi A., Syrigos K.N. (2016). The use of novel oral anticoagulants in cancer patients with venous thromboembolism. Semin. Oncol..

[65] Wang C.X., Wu D., Yang P.P., Wu Q.H. (2020). Efficacy and safety of non-vitamin K antagonist versus vitamin K antagonist oral anticoagulants in the prevention and treatment of thrombotic disease in active cancer patients: A systematic review and meta-analysis of randomized controlled trials. Zhonghua Xin Xue Guan Bing Za Zhi.

[66] Verso M., Agnelli G., Prandoni P. (2015). Pros and cons of new oral anticoagulants in the treatment of venous thromboembolism in patients with cancer. Intern. Emerg. Med..

[67] Haas S., Farjat A.E., Pieper K., Ageno W., Angchaisuksiri P., Bounameaux H., Goldhaber S.Z., Goto S., Mantovani L., Prandoni P. (2022). On-treatment Comparative Effectiveness of Vitamin K Antagonists and Direct Oral Anticoagulants in GARFIELD-VTE, and Focus on Cancer and Renal Disease. TH Open.

[68] Barbarawi M., Barbarawi O., Corcoran J., Obeidat K., Al-Abdouh A., Mhanna M., Al Kasasbeh M., Pickett C.C. (2022). Efficacy and Safety of the Non-Vitamin K Antagonist Oral Anticoagulant Among Patients with Nonvalvular Atrial Fibrillation and Cancer: A Systematic Review and Network Meta-analysis. Curr. Probl. Cardiol..

[69] Chan Y.H., Chao T.F., Lee H.F., Chen S.W., Li P.R., Liu J.R., Wu L.S., Chang S.H., Yeh Y.H., Kuo C.T. (2021). Clinical Outcomes in Atrial Fibrillation Patients with a History of Cancer Treated with Non-Vitamin K Antagonist Oral Anticoagulants: A Nationwide Cohort Study. Stroke.

[70] Clemens A., Strack A., Noack H., Konstantinides S., Brueckmann M., Lip G.Y. (2014). Anticoagulant-related gastrointestinal bleeding--could this facilitate early detection of benign or malignant gastrointestinal lesions?. Ann. Med..

[71] Papanastasiou A., Morsi-Yeroyannis A., Karagiannidis E., Kartas A., Doundoulakis I., Karvounis H., Giannakoulas G. (2021). Association of anticoagulant-related bleeding events with cancer detection in atrial fibrillation: A systematic review and meta-analysis. Hell. J. Cardiol..

[72] Chang T.Y., Chan Y.H., Chiang C.E., Lin Y.J., Chang S.L., Lo L.W., Hu Y.F., Tuan T.C., Liao J.N., Chung F.P. (2020). Risks and outcomes of gastrointestinal malignancies in anticoagulated atrial fibrillation patients experiencing gastrointestinal bleeding: A nationwide cohort study. Heart Rhythm..

[73] Antunes L.F. (2020). New Oral Anticoagulants (NOACs) are The Gold Standard Invenous Thromboembolism. Rev. Port. Cir. Cardiotorac. Vasc..

[74] Choi Y.J., Choi Y.W., Chae J.W., Yun H.Y., Shin S. (2021). Clinical Benefits of Oral Anticoagulant Use in Cancer Patients at Increased Risk for Venous Thromboembolism per Khorana Index. Risk Manag. Healthc. Policy.

[75] Chen Y., Mao M., Chang J., Yan J., Yang T., Liu Y., Luo M., Hu Y., Yang Q., Zhou L. (2021). Safety and efficacy of new oral anticoagulants compared to those of warfarin in AF patients with cancer: A meta-analysis of randomized clinical trials and observational studies. Eur. J. Clin. Pharmacol..

[76] Wu V.C., Wang C.L., Huang Y.T., Lan W.C., Wu M., Kuo C.F., Chen S.W., Chu P.H., Wen M.S., Kuo C.C. (2020). Novel Oral Anticoagulant versus Warfarin in Cancer Patients with Atrial Fibrillation: An 8-Year Population-Based Cohort Study. J. Cancer.

[77] Kim K., Lee Y.J., Kim T.H., Uhm J.S., Pak H.N., Lee M.H., Joung B. (2018). Effect of Non-vitamin K Antagonist Oral Anticoagulants in Atrial Fibrillation Patients with Newly Diagnosed Cancer. Korean Circ. J..

[78] Atterman A., Asplund K., Friberg L., Engdahl J. (2020). Use of oral anticoagulants after ischemic stroke in patients with atrial fibrillation and cancer. J. Intern. Med..

[79] Solari F., Varacallo M. (2018). Low Molecular Weight Heparin (LMWH).

[80] Mulloy B., Hogwood J., Gray E., Lever R., Page C.P. (2016). Pharmacology of Heparin and Related Drugs. Pharmacol. Rev..

[81] Howard L.S. (2018). Non-vitamin K antagonist oral anticoagulants for pulmonary embolism: Who, where and for how long?. Expert Rev. Respir. Med..

[82] Short N.J., Connors J.M. (2014). New oral anticoagulants and the cancer patient. Oncologist.

[83] Enea I., Roncon L., Gulizia M.M., Azzarito M., Becattini C., Bongarzoni A., Casazza F., Cuccia C., D’Agostino C., Rugolotto M. (2017). ANMCO Position Paper: The use of non-vitamin K dependent new oral anticoagulant(s) in pulmonary embolism therapy and prevention. Eur. Heart J. Suppl..

[84] Al-Samkari H., Connors J.M. (2018). The Role of Direct Oral Anticoagulants in Treatment of Cancer-Associated Thrombosis. Cancers.

[85] Stepien K., Nowak K., Zalewski J., Pac A., Undas A. (2019). Extended treatment with non-vitamin K antagonist oral anticoagulants versus low-molecular-weight heparins in cancer patients following venous thromboembolism. A pilot study. Vascul. Pharmacal..

[86] Tirandi A., Preda A., Carbone F., Montecucco F., Liberale L. (2022). Pulmonary embolism in patients with cancer: An updated and operative guide for diagnosis and management. Int. J. Cardiol..

[87] Asnani A., Manning A., Mansour M., Ruskin J., Hochberg E.P., Ptaszek L.M. (2017). Management of atrial fibrillation in patients taking targeted cancer therapies. Cardiooncology.

[88] Jin C., Cui C., Seplowe M., Lee K.I., Vegunta R., Li B., Frishman W.H., Iwai S. (2022). Anticoagulation for Atrial Fibrillation: A Review of Current Literature and Views. Cardiol. Rev..

[89] Stevens S.M., Woller S.C., Kreuziger L.B., Bounameaux H., Doerschug K., Geersing G.J., Huisman M.V., Kearon C., King C.S., Knighton A.J. (2021). Antithrombotic therapy for VTE disease: Second update of the CHEST guideline and expert panel report. Chest.

[90] Gressel G.M., Marcus J.Z., Mullen M.M., Sinno A.K. (2021). Direct oral anticoagulant use in gynecologic oncology: A Society of Gynecologic Oncology Clinical Practice Statement. Gynecol. Oncol..

[91] Ren J.W., Zheng X., Han X.H. (2023). Generic Methods for Simultaneous Analysis of Four Direct Oral Anticoagulants in Human Plasma and Urine by Ultra-High Performance Liquid Chromatography-Tandem Mass Spectrometry. Molecules.

[92] Lenihan D.J., Fradley M.G., Dent S., Brezden-Masley C., Carver J., Filho R.K., Neilan T.G., Blaes A., Melloni C., Herrmann J. (2019). Proceedings from the Global Cardio-Oncology Summit: The Top 10 Priorities to Actualize for CardioOncology. JACC CardioOncol..

[93] Khorana A.A., Kuderer N.M., Culakova E., Lyman G.H., Francis C.W. (2008). Development and validation of a predictive model for chemotherapy-associated thrombosis. Blood.

[94] Key N.S., Khorana A.A., Kuderer N.M., Bohlke K., Lee A.Y.Y., Arcelus J.I., Wong S.L., Balaban E.P., Flowers C.R., Francis C.W. (2020). Venous Thromboembolism Prophylaxis and Treatment in Patients with Cancer: ASCO Clinical Practice Guideline Update. J. Clin. Oncol..

[95] Falanga A., Ay C., Di Nisio M., Gerotziafas G., Langer F., Lecumberri R., Mandala M., Maraveyas A., Pabinger I., Jara-Palomares L. (2023). Venous thromboembolism in cancer patients: ESMO Clinical Practice Guideline. Ann. Oncol..

[96] Streiff M.B., Holmstrom B., Angelini D., Ashrani A., Elshoury A., Fanikos J., Fertrin K.Y., Fogerty A.E., Gao S., Goldhaber S.Z. (2021). Cancer-Associated Venous Thromboembolic Disease, Version 2.2021, NCCN Clinical Practice Guidelines in Oncology. J. Natl. Compr. Cancer Netw..

[97] Lyman G.H., Carrier M., Ay C., Di Nisio M., Hicks L.K., Khorana A.A., Leavitt A.D., Lee A.Y.Y., Macbeth F., Morgan R.L. (2021). American Society of Hematology 2021 guidelines for management of venous thromboembolism: Prevention and treatment in patients with cancer. Blood Adv..

[98] Li M., Li J., Wang X., Hui X., Wang Q., Xie S., Yan P., Tian J., Li J., Xie P. (2023). Oral direct thrombin inhibitors or oral factor Xa inhibitors versus conventional anticoagulants for the treatment of pulmonary embolism. Cochrane Database Syst. Rev..

[99] Barca-Hernando M., Lopez-Ruz S., Marin-Romero S., Garcia-Garcia V., Elias-Hernandez T., Otero-Candelera R., Carrier M., Jara-Palomares L. (2023). Risk of recurrent cancer-associated thrombosis after discontinuation of anticoagulant therapy. Res. Pract. Thromb. Haemost..

[100] Debourdeau P., Bertoletti L., Font C., López-Núñez J.J., Gómez-Cuervo C., Mahe I., Otero-Candelera R., Adarraga M.D., López-Miguel P., Monreal M. (2023). Three-Month Outcomes in Cancer Patients with Superficial or Deep Vein Thrombosis in the Lower Limbs: Results from the RIETE Registry. Cancers.

[101] Robinson R., Spectre G., Lishner M., Sharabi O., Robinson E., Hamburger Avnery O., Gafter-Gvili A., Raanani P., Leader A. (2023). Direct oral anticoagulants in patients with venous thromboembolism and hematological malignancies. J. Thromb. Thrombolysis.

[102] Wiklund P., Medson K., Elf J. (2023). Unreported incidental pulmonary embolism in patients with cancer: Radiologic natural history and risk of recurrent venous thromboembolism and death. Thromb. Res..

[103] Vedovati M.C., Giustozzi M., Munoz A., Bertoletti L., Cohen A.T., Klok F.A., Connors J.M., Bauersachs R., Brenner B., Campanini M. (2023). Risk factors for recurrence and major bleeding in patients with cancer-associated venous thromboembolism. Eur. J. Intern. Med..

[104] Li A., Garcia D.A., Lyman G.H., Carrier M. (2019). Direct oral anticoagulant (DOAC) versus low-molecular-weight heparin (LMWH) for treatment of cancer associated thrombosis (CAT): A systematic review and meta-analysis. Thromb. Res..

[105] Lv S., Liu Y., Wei G., Shi X., Chen S., Zhang X. (2019). The anticoagulants rivaroxaban and low molecular weight heparin prevent PICC-related upper extremity venous thrombosis in cancer patients. Medicine.

[106] Longo de Oliveira A.L.M., de Oliveira Pereira R.F., Agati L.B., Ribeiro C.M., Kawamura Suguiura G.Y., Cioni C.H., Bermudez M., Pirani M.B., Caffaro R.A., Castelli V. (2022). Rivaroxaban Versus Enoxaparin for Thromboprophylaxis After major Gynecological Cancer Surgery: The VALERIA Trial: Venous thromboembolism prophylAxis after gynecoLogical pElvic cancer surgery with RIvaroxaban versus enoxAparin (VALERIA trial). Clin. Appl. Thromb. Hemost..

[107] Chen D.Y., Tseng C.N., Hsieh M.J., Lan W.C., Chuang C.K., Pang S.T., Chen S.W., Chen T.H., Chang S.H., Hsieh I.C. (2021). Comparison between Non-vitamin K Antagonist Oral Anticoagulants and Low-Molecular-Weight Heparin in Asian Individuals With Cancer-Associated Venous Thromboembolism. JAMA Netw. Open.

[108] Song A.B., Rosovsky R.P., Connors J.M., Al-Samkari H. (2019). Direct oral anticoagulants for treatment and prevention of venous thromboembolism in cancer patients. Vasc. Health Risk Manag..

[109] Camilli M., Lombardi M., Vescovo G.M., Del Buono M.G., Galli M., Aspromonte N., Zoccai G.B., Niccoli G., Montone R.A., Crea F. (2020). Efficacy and safety of novel oral anticoagulants versus low molecular weight heparin in cancer patients with venous thromboembolism: A systematic review and meta-analysis. Crit. Rev. Oncol. Hematol..

[110] Raskob G.E., van Es N., Verhamme P., Carrier M., Di Nisio M., Garcia D., Grosso M.A., Kakkar A.K., Kovacs M.J., Mercuri M.F. (2018). Edoxaban for the Treatment of Cancer-Associated Venous Thromboembolism. N. Engl. J. Med..

[111] Wang K.L., Kao Y.T., Chang W.T., Chang H.Y., Huang W.C., Hsu P.C., Hsu C.H., Huang C.L., Hsieh L.C., Wang C.Y. (2020). Management of Venous Thromboembolisms: Part, I.I. The Consensus for Pulmonary Embolism and Updates. Acta Cardiol. Sin..

[112] Hussain M.R., Ali F.S., Verghese D., Myint P.T., Ahmed M., Gong Z., Gerais Y., Siddiqui M., Lin J.J., Troy K. (2022). Factor Xa inhibitors versus low molecular weight heparin for the treatment of cancer associated venous thromboembolism; A meta-analysis of randomized controlled trials and non-randomized studies. Crit. Rev. Oncol. Hematol..

[113] Frere C., Farge D., Schrag D., Prata P.H., Connors J.M. (2022). Direct oral anticoagulant versus low molecular weight heparin for the treatment of cancer-associated venous thromboembolism: 2022 updated systematic review and meta-analysis of randomized controlled trials. J. Hematol. Oncol..

[114] Nam K.W., Kim C.K., Kim T.J., An S.J., Oh K., Ko S.B., Yoon B.W. (2017). Treatment of Cryptogenic Stroke with Active Cancer with a New Oral Anticoagulant. J. Stroke Cerebrovasc. Dis..

[115] Jiménez-Fonseca P., Gallardo E., Arranz Arija F., Blanco J.M., Callejo A., Lavin D.C., Costa Rivas M., Mosquera J., Rodrigo A., Sánchez Morillas R. (2022). Consensus on prevention and treatment of cancer-associated thrombosis (CAT) in controversial clinical situations with low levels of evidence. Eur. J. Intern. Med..

[116] Delluc A., Wang T.F., Yap E.S., Ay C., Schaefer J., Carrier M., Noble S. (2019). Anticoagulation of cancer patients with non-valvular atrial fibrillation receiving chemotherapy: Guidance from the SSC of the ISTH. J. Thromb. Haemost..

[117] Undas A., Drabik L. (2020). Non-vitamin K antagonist oral anticoagulants (NOACs) in cancer patients with atrial fibrillation. Anatol. J. Cardiol..

[118] Ferri N., Colombo E., Tenconi M., Baldessin L., Corsini A. (2022). Drug-Drug Interactions of Direct Oral Anticoagulants (DOACs): From Pharmacological to Clinical Practice. Pharmaceutics.

[119] Otten L.S., Piet B., van den Heuvel M.M., Marzolini C., van Geel R.M.J.M., Gulikers J.L., Burger D.M., Leentjens J., Ter Heine R. (2022). Practical recommendations to combine small-molecule inhibitors and direct oral anticoagulants in patients with nonsmall cell lung cancer. Eur. Respir. Rev..

[120] Hellfritzsch M., Henriksen J.N., Holt M.I., Grove E.L. (2023). Drug-Drug Interactions in the Treatment of Cancer-Associated Venous Thromboembolism with Direct Oral Anticoagulants. Seminars in Thrombosis and Hemostasis.

[121] Rousseau A., Van Dreden P., Mbemba E., Elalamy I., Larsen A., Gerotziafas G.T. (2015). Cancer cells BXPC3 and MCF7 differentially reverse the inhibition of thrombin generation by apixaban, fondaparinux and enoxaparin. Thromb. Res..

[122] Muñoz Martín A.J., Gallardo Díaz E., García Escobar I., Macías Montero R., Martínez-Marín V., Pachón Olmos V., Pérez Segura P., Quintanar Verdúguez T., Salgado Fernández M. (2020). SEOM clinical guideline of venous thromboembolism (VTE) and cancer (2019). Clin. Transl. Oncol..

[123] Tran H.A., Gibbs H., Merriman E., Curnow J.L., Young L., Bennett A., Tan C.W., Chunilal S.D., Ward C.M., Baker R. (2019). New guidelines from the Thrombosis and Haemostasis Society of Australia and New Zealand for the diagnosis and management of venous thromboembolism. Med. J. Aust..

[124] Nayak A.L., Zahrai A., Mallick R., Wang T.F., Delluc A., Castellucci L.A., Carrier M., Wells P.S. (2021). Efficacy of primary prevention of venous thromboembolism among subgroups of cancer patients undergoing chemotherapy: A post- hoc analysis of the AVERT trial. Thromb. Res..

[125] Ladha D., Mallick R., Wang T.F., Caiano L., Wells P.S., Carrier M. (2021). Efficacy and safety of apixaban for primary prevention in gastrointestinal cancers: A post-hoc analysis of the AVERT trial. Thromb. Res..

[126] Brandt W., Brown C., Wang T.F., Tagalakis V., Shivakumar S., Ciuffini L.A., Mallick R., Wells P.S., Carrier M. (2022). Efficacy and safety of apixaban for primary prevention of thromboembolism in patients with cancer and a central venous catheter: A subgroup analysis of the AVERT Trial. Thromb. Res..

[127] Wang T.F., Mallick R., Carrier M., Wells P.S. (2022). Safety and efficacy of apixaban thromboprophylaxis in ambulatory cancer patients according to renal function: A subgroup analysis of the AVERT trial. Thromb. Res..

[128] Knoll W., Mallick R., Wells P.S., Carrier M. (2021). Safety and efficacy of apixaban thromboprophylaxis in cancer patients with metastatic disease: A post-hoc analysis of the AVERT trial. Thromb. Res..

[129] Zhang J., Atalla M., Mallick R., Wells P.S., Carrier M. (2021). Thromboprophylaxis for patients with newly diagnosed vs. recurrent cancers: A post-hoc analysis of the avert trial. J. Thromb. Thrombolysis.

[130] Mones J.V., Streiff M.B., Khorana A.A., Bendheim G.A., Damaraju C.V., Wildgoose P., Burton P., Riess H., Soff G.A. (2021). Rivaroxaban thromboprophylaxis for gastric/gastroesophageal junction tumors versus other tumors: A post hoc analysis of the randomized CASSINI trial. Res. Pract. Thromb. Haemost..

[131] Khorana A.A., McNamara M.G., Kakkar A.K., Streiff M.B., Riess H., Vijapurkar U., Kaul S., Wildgoose P., Soff G.A., CASSINI Investigators (2020). Assessing Full Benefit of Rivaroxaban Prophylaxis in High-Risk Ambulatory Patients with Cancer: Thromboembolic Events in the Randomized CASSINI Trial. TH Open.

[132] Vadhan-Raj S., McNamara M.G., Venerito M., Riess H., O’Reilly E.M., Overman M.J., Zhou X., Vijapurkar U., Kaul S., Wildgoose P. (2020). Rivaroxaban thromboprophylaxis in ambulatory patients with pancreatic cancer: Results from a pre-specified subgroup analysis of the randomized CASSINI study. Cancer Med..

[133] Khorana A.A., Soff G.A., Kakkar A.K., Vadhan-Raj S., Riess H., Wun T., Streiff M.B., Garcia D.A., Liebman H.A., Belani C.P. (2019). Rivaroxaban for Thromboprophylaxis in High-Risk Ambulatory Patients with Cancer. N. Engl. J. Med..

[134] National Library of Medicine (U.S.) (2021). Interaction between Direct Oral Anticoagulants and Drug-Metabolizing Enzyme Inducers. Identifier: NCT05750680. NCT05750680.

[135] National Library of Medicine (U.S.) (2019). Clinical Application Model of Direct Oral Anticoagulants (MACACOD). Comprehensive Management of ACOD from a Specialized Center in Antithrombotic Therapy and Its Area of Influence Identifier: NCT04042155. NCT04042155.

[136] National Library of Medicine (U.S.) (2016). Direct Oral Anticoagulants (DOACs) Versus LMWH +/- Warfarin for VTE in Cancer: A Randomized Effectiveness Trial (CANVAS Trial). Identifier: NCT02744092. NCT02744092.

[137] National Library of Medicine (U.S.) VICTORIE (VTE In Cancer—Treatment, Outcomes and Resource Use in Europe). Identifier: NCT04618913. NCT04618913.

[138] Castle J., Blower E., Bundred N.J., Harvey J.R., Thachil J., Marshall A., Cox K., Cicconi S., Holcombe C., Palmieri C. (2020). Rivaroxaban compared to no treatment in ER-negative stage I-III early breast cancer patients (the TIP Trial): Study protocol for a phase II preoperative window-of-opportunity study design randomised controlled trial. Trials.

[139] National Library of Medicine (U.S.) (2022). Observational Studies in Cancer Associated Thrombosis for Rivaroxaban in Sweden (OSCAR-SE). Identifier: NCT05150938. NCT05150938.

[140] National Library of Medicine (U.S.) (2022). Primary Thromboprophylaxis in Patients with Malignancy and Central Venous Catheters: A Randomized Controlled Trial. Identifier: NCT05029063. NCT05029063.

[141] National Library of Medicine (U.S.) (2021). Bleeding Risk Guided VTE Prophylaxis Strategy for Hospitalized Patients with Lung Cancer: Rationale and Design for a Multicenter, Adjudicator-Blinded, Parallel, Randomized Clinical Trial in China. Identifier: NCT04158973. NCT04158973.

[142] National Library of Medicine (U.S.) (2023). Venous Thromboembolism Prevention in Outpatients with Glioma. Identifier: NCT05683808. NCT05683808.

[143] National Library of Medicine (U.S.) (2022). Cost Comparison between Apixaban and Low Molecular Weight Heparin (LMWH) among Venous Thromboembolism (VTE) Cancer Patients. Identifier: NCT05643885. NCT05643885.

[144] National Library of Medicine (U.S.) (2022). A Multicenter, Randomized, Open-Label, Blinded Endpoint Evaluation, Phase 3 Study Comparing the Effect of Abelacimab Relative to Apixaban on Venous Thromboembolism (VTE) Recurrence and Bleeding in Patients with Cancer Associated VTE. Identifier: NCT05171049. NCT05171049.

[145] Planquette B., Bertoletti L., Charles-Nelson A., Laporte S., Grange C., Mahé I., Pernod G., Elias A., Couturaud F., Falvo N. (2022). Rivaroxaban vs. Dalteparin in Cancer-Associated Thromboembolism: A Randomized Trial. Chest.

[146] Agnelli G., Becattini C., Meyer G., Muñoz A., Huisman M.V., Connors J.M., Cohen A., Bauersachs R., Brenner B., Torbicki A. (2020). Apixaban for the Treatment of Venous Thromboembolism Associated with Cancer. N. Engl. J. Med..

[147] Canonico M.E., Santoro C., Avvedimento M., Giugliano G., Mandoli G.E., Prastaro M., Franzone A., Piccolo R., Ilardi F., Cameli M. (2022). Venous Thromboembolism and Cancer: A Comprehensive Review from Pathophysiology to Novel Treatment. Biomolecules.

